# Cellular Internalization and Toxicity of Chitosan Nanoparticles Loaded with Nobiletin in Eukaryotic Cell Models (*Saccharomyces cerevisiae* and *Candida albicans*)

**DOI:** 10.3390/ma17071525

**Published:** 2024-03-27

**Authors:** Pedro Amado Hernández-Abril, Ana Karenth López-Meneses, Jaime Lizardi-Mendoza, Maribel Plascencia-Jatomea, Ana Guadalupe Luque-Alcaraz

**Affiliations:** 1 Departamento de Ingeniería Biomédica, Universidad Estatal de Sonora, Hermosillo 83100, Sonora, Mexico; 2 Microbiology and Mycotoxins Laboratory, Departamento de Investigación y Posgrado en Alimentos, Universidad de Sonora, Hermosillo 83000, Sonora, Mexico; anakarenth.lopez@unison.mx (A.K.L.-M.); maribel.plascencia@unison.mx (M.P.-J.); 3 Biopolymer Laboratory, Centro de Investigación y Desarrollo en Alimentación, A.C., Hermosillo 83304, Sonora, Mexico; jalim@ciad.mx

**Keywords:** chitosan, nobiletin, cellular, nanoparticles

## Abstract

This study involved the synthesis and characterization of chitosan nanoparticles loaded with nobiletin (CNpN) and assessed their toxicity and cellular internalization in eukaryotic cell models (*Saccharomyces cerevisiae* and *Candida albicans*). Nanoparticles were prepared via the nanoprecipitation method and physicochemically characterized to determine their hydrodynamic diameter using dynamic light scattering (DLS), their surface charge through ζ-potential measurements, and their chemical structure via Fourier-transform infrared spectroscopy (FTIR). The hydrodynamic diameter and ζ-potential of chitosan nanoparticles (CNp) and CNpN were found to be 288.74 ± 2.37 nm and 596.60 ± 35.49 nm, and 34.51 ± 0.66 mV and 37.73 ± 0.19 mV, respectively. The scanning electron microscopy (SEM) images displayed a particle size of approximately 346 ± 69 nm, with notable sphericity for CNpN. FTIR analysis provided evidence of potential imine bonding between chitosan and nobiletin. Membrane integrity damage could be observed in both *S. cerevisiae* and *C. albicans* yeast stained with propidium iodide, demonstrating membrane integrity damage caused by CNp and CNpN, where higher concentration treatments inhibited the development of yeast cells. These findings suggest a selective therapeutic potential of CNpN, which could be promising for the development of antifungal and anticancer therapies. This study contributes to understanding the interaction between nanoparticles and eukaryotic cells, offering insights for future biomedical applications.

## 1. Introduction

In the constant quest for more effective and specific therapies to address a wide range of diseases, nanotechnology has emerged as a highly interesting field in the biomedical domain [1]. The ability to design and manipulate materials at the nanoscale has revolutionized the development and administration of medical treatments, offering new insights into enhancing therapeutic efficacy while reducing unwanted side effects [2]. Within this context, CNp have gained prominence due to their versatility, biocompatibility, and capacity to be functionalized with various therapeutic agents [3]. Chitosan, a natural polysaccharide derived from chitin, has been extensively studied for its potential application in controlled drug delivery [4]. Its unique chemical structure and favorable biological properties make it an attractive candidate for the formulation of drug delivery systems [5].

Among the different flavonoids, nobiletin has emerged as a compound of special interest due to its broad spectrum of pharmacological activities, including its anti-inflammatory [6], antioxidant [7], neuroprotective [8], and anticancer properties [9]. This flavonoid is primarily found in citrus fruits, such as oranges and tangerines, and has demonstrated beneficial effects in a variety of experimental models and clinical studies [10,11]. The ability of nobiletin to modulate multiple cellular pathways makes it a potentially valuable therapeutic agent for the treatment of chronic diseases, such as Alzheimer’s disease [12], cancer [9], and cardiovascular diseases [13].

In the context of nanomedicine, the combination of the beneficial properties of chitosan and nobiletin in the form of nanoparticles offers an innovative strategy to enhance the therapeutic efficacy of this flavonoid. Previous studies have explored the possibility of encapsulating nobiletin in CNp as an approach to improve its solubility, stability, and bioavailability [14]. Encapsulation in CNp may also provide a means to achieve the controlled release of nobiletin at the desired site of action, potentially reducing systemic toxicity and improving therapeutic efficacy. However, there are still questions regarding the mechanisms underlying the cellular internalization of these nanoparticles and their influence on the biological activity of nobiletin.

The cellular internalization of nanoparticles is a complex process that may be influenced by various factors, including size, shape, surface charge, and chemical composition of the particles [15]. Understanding how CNp loaded with nobiletin are internalized by eukaryotic cells is crucial for optimizing their therapeutic efficacy and minimizing potential adverse effects. In this context, the present study focused on the synthesis and characterization of (CNpN) and the evaluation of their toxicity and cellular internalization capacity in eukaryotic cell models, specifically *S. cerevisiae* and *C. albicans*. These cellular models have been widely used in biological research due to their simplicity and relevance in the study of fundamental cellular processes [16,17].

The plasma membrane constitutes an integral component of all living cells, fulfilling a pivotal role in myriad physiological processes, including material exchange, stress response, cellular recognition, signal transduction, cellular immunity, apoptosis, and pathogenicity [18]. Additionally, it maintains the physical and structural integrity of the cell.

The use of distinct fluorescent biomarkers for cellular identification enables the microscopic examination of various indicators of cell damage, including plasma membrane integrity, fragmented apoptotic cells or nuclei, and uptake of fragmented cells by neighboring cells. Cellular damage typically manifests through observable signs of necrosis or apoptosis in fluorescence images, such as cell swelling, indiscriminate fragmentation, cell contraction, and condensation, or the nonrandom fragmentation of genetic material [19].

The membranes of living cells are impermeable to propidium iodide, whereas only damaged and/or altered cells can be stained, as propidium iodide penetrates the cell interior, reacts, and intercalates with 4–5 base pairs of DNA, emitting high red fluorescence [19,20].

Recent investigations have examined membrane damage in various fungi using nanoparticles composed of diverse materials. Kiefer et al. (2019) [21] synthesized poly(lactic-co-glycolic acid) (PLGA) nanoparticles to evaluate their effect on *S. cerevisiae*, developing nanoparticles/yeast complexes that enabled compound association and even internalization. Under hypotonic conditions, 90% of the yeast cells stained red with propidium iodide, confirming membrane permeability under such exposure. In contrast, it has been reported that magnesium peroxide nanoparticles increase membrane permeability in *Alternaria alternata*, eventually causing spore rupture and death.

Regarding chitosan nanomaterials, studies report that CNp with silver impair membrane integrity in *C. albicans*, attributing this effect to the positive charges of the amino groups (NH_3_^+^) in chitosan that attract the negative charges on the fungal cell surface, causing alteration and leakage of intracellular content or cell death [20]. Similarly, observed damage to *C. albicans* when exposed to varying concentrations of chitosan–gold nanocomposites, with membrane permeability damage greater than the minimum fungicidal concentration (MFC) of 75 μg/mL [20].

This study is based on a broad body of previous research supporting the use of CNp for the delivery of therapeutic agents [5,22]. Additionally, numerous studies have demonstrated the potential benefits of nobiletin in the treatment of various diseases, supporting its inclusion in this research [11,23,24]. The combination of these two approaches in the form of CNpN represents a logical extension of previous investigations and has the potential to yield significant results in terms of therapeutic efficacy and selectivity. However, addressing the gaps in our knowledge regarding the mechanisms of cellular internalization of these nanoparticles is important, as this may influence their therapeutic efficacy and safety.

Together, the results of this study provide crucial information on the interaction between CNpN and eukaryotic cells, contributing to the optimization of nanotechnology-based therapeutic strategies for disease treatment. Furthermore, these findings could open new avenues of research in the field of nanomedicine and facilitate the development of more effective and safer therapies to improve human health.

## 2. Materials and Methods

### 2.1. Materials

Materials used in this study included medium-molecular-weight commercial chitosan (448877; Sigma-Aldrich, St. Louis, MO, USA), XTT salt (Sigma-Aldrich) (2,3-Bis(2-methoxy-4-nitro-5-sulfophenyl)-2H-tetrazolium-5-carboxanilide inner salt), methanol (Sigma-Aldrich), and nobiletin acquired from SCBT (Dallas, TX, USA) (CAS 478-01-3, purity: >98%), potato dextrose broth (PDB) acquired from Difco^TM^ (Franklin Lakes, NJ, USA). All other reagents and solvents (Sigma-Aldrich) were utilized without further purification. Experiments were conducted using distilled water. The peristaltic pump utilized was model 73160-31 (Cole-Parmer, Vernon Hills, IL, USA).

### 2.2. Synthesis of CNp

The nanoparticles were prepared using the nanoprecipitation method according to the procedure outlined in previous research [14]. A solution of chitosan with 1% acetic acid at a concentration of 10 mg/mL was prepared. From this solution, 2.5 mL was taken to form the solvent phase and added to the nonsolvent phase consisting of 40 mL of methanol. This process was carried out by subjecting the nonsolvent phase to magnetic stirring at 500 rpm and adding the solvent phase dropwise at a flow rate of 0.60 mL/min supplied by a peristaltic pump, forming the CNp. Subsequently, the methanol was recovered using a rotary evaporator. For the synthesis of CNpN, the same method was used, incorporating nobiletin into the nonsolvent phase.

### 2.3. DLS and ζ-Potential

The hydrodynamic diameter and ζ-potential were determined through dynamic light scattering analysis using a Möbiuz (Wyatt Technology, Santa Barbara, CA, USA). A volume of 90 µL was introduced into the instrument’s sample cell, which was equipped with a vertically polarized laser emitting at a wavelength of 488 nm (2W). The detection angle was fixed at 90° relative to the incident light beam. Three replicates were conducted, each lasting 60 s, at ambient temperature. The average values were calculated from these replicates to ensure statistical robustness [25].

### 2.4. Fourier-Transform Infrared Spectroscopy (FTIR)

The interaction between chitosan and nobiletin was investigated using Fourier-transform infrared spectroscopy (FTIR). Spectra were acquired using a Perkin–Elmer FTIR Spectrum GX instrument (Shelton, CT, USA), averaging 16 scans over a spectral range from 1700 to 1500 cm^−1^. The analysis was conducted in a liquid medium.

### 2.5. Scanning Electron Microscopy (SEM)

A total of 5 mL of each sample was transferred into a 10 mL vial and subjected to sonication for 20 min at 60 °C. Subsequently, three drops of the sonicated samples were applied onto the sample holder, utilizing highly conductive graphite paint as an adhesive. The samples were left to air dry for 24 h. The morphology of the dried samples was examined via scanning electron microscopy using a JEOL 6360LV microscope (JEOL Inc., Peabody, MA, USA) operated under high-vacuum conditions, with settings at 20 kV and a spot size of 11.

### 2.6. Inoculum Reactivation

Two strains of yeast, *S. cerevisiae* (ATCC 9763) and *C. albicans* (ATCC 14053), were reactivated in PDB, incubating them for 48 h at 37 ± 2 °C. Following the incubation period, a yeast suspension was prepared, counting 4 × 10^6^ yeast/mL, using a Neubauer chamber.

### 2.7. Nanoparticles Treatment Preparation

To prepare the treatments, a serial dilution of CNp and CNpN was performed to obtain five different concentrations (A, B, C, D, and Control), as shown in Table 1.

### 2.8. Cell Viability

The colorimetric assay for quantifying yeast viability was conducted following the method described by Luque-Alcaraz et al. (2016) [25]. A solution of the XTT tetrazolium salt was prepared at a concentration of 2 mg/mL in saline solution and passed through a filter with a pore size of 0.2 μm. Additionally, a menadione solution in acetone was prepared at a concentration of 10 mM, which was diluted to 1 mM by taking a 1 mL aliquot and homogenizing it with 9 mL of saline solution. In a 96-well microplate, 100 μL of inoculum (4 × 10^6^ yeast/mL) was dispensed and incubated for 4 h at 37 ± 2 °C. Subsequently, nanoparticles and controls at different concentrations were applied to treatments A, B, C, and D, depositing 100 μL in each well, and the microplate was incubated again for another 4h. The treatment referred to as the Control is the PDB where the yeasts could grow freely without any interference, and the control was be used as a reference. After this period, 50 μL of the XTT solution and 7 μL of the menadione solution were added to each well, and it was further incubated for 3 h. Finally, the absorbance of each well in the microplate was read using an ELISA spectrophotometer (800 TS microplate reader) at a wavelength of 450 nm from Agilent (Santa Clara, CA, USA). The percentage of cell viability was determined relative to the control.

### 2.9. Membrane Permeability Damage

Propidium iodide is a fluorescent stain for nucleic acids and cell membrane integrity and excludes PI from staining viable and apoptotic or damaged cells. In a 96-well microplate, 100 μL of yeast suspension at a concentration of 2 × 10^6^ yeast/mL in PDB was dispensed and incubated at 37 ± 2 °C for 3 h. After this incubation period, 100 μL of different nanoparticles and controls was added and incubated for 24 h under the same temperature conditions. Following the 24 h incubation, 10 μL of 3 μM propidium iodide (PI) was added, and the samples were again incubated at the same temperature for 1 h. After incubation, cells were analyzed using a fluorescence microscope (Model DMi8; Leica Microsystems, Wetzlar, Germany) equipped with fluorescence filters (546/10 excitation filter for PI and 585/40 emission filter), a cooled monochromatic DFC450 C camera (Leica Microsystems, Wetzlar, Germany), and fluorescence overlay software (LAS AF ver. 3.1.0; Leica Microsystems CMS GmbH, Mannheim, Germany).

### 2.10. Experimental Design and Statistical Analysis

A completely randomized experimental design was utilized, comprising each of the treatments (CNp and CNpN) at different concentrations (A, B, C, and D), as well as nobiletin in solution as control. We analyzed the differences between each of the concentrations within each treatment. The experimental data were analyzed by means of one-way analysis of variance (ANOVA) employing NCSS 97 software (NCSS, Inc., Kaysville, UT, USA) at a significance level of *p* = 0.05. The means analysis was carried out through a multivariate range Tukey test (Tukey post hoc test) at a 95% confidence interval. All the results are presented with mean ± standard error.

## 3. Results

### 3.1. Hydrodynamic Diameter and ζ-Potential Analysis of CNp and CNpN

The hydrodynamic diameter and ζ-potential of CNp and CNpN were determined. CNp exhibited a hydrodynamic diameter of 288.74 ± 2.37 nm, while CNpN showed a larger diameter of 596.60 ± 35.49 nm. Additionally, the ζ-potential of CNp was measured at 34.51 ± 0.66 mV, whereas CNpN exhibited a slightly higher ζ-potential of 37.73 ± 0.19 mV (Table 2). The positive ζ-potential of both nanoparticle formulations indicates their potential for colloidal stability and electrostatic repulsion, which could prevent aggregation and enhance their dispersibility in biological systems. Moreover, the larger hydrodynamic diameter of CNpN suggested the successful loading of nobiletin onto the CNp. This increase in size could be attributed to the incorporation of nobiletin molecules into the chitosan matrix. The obtained sizes and ζ-potentials are comparable to those reported in a previous study, indicating the reproducibility and reliability of the synthesis method employed in this study. The increases in hydrodynamic diameter and surface charge coincides with those reported in a previous study in which the same methodology and molar ratios were used. In that study, the association and loading efficiencies were determined to be 69.1% and 7.0%, respectively [14]. The larger size of CNpN may offer advantages in terms of drug loading capacity and sustained release profiles, which could be beneficial for targeted drug delivery applications. Furthermore, the positive ζ-potential of both CNp and CNpN suggests their potential for use in various biomedical applications, including drug delivery, imaging, and theragnostics.

### 3.2. SEM Characterization and Morphological Analysis of CNp and CNpN

**Figure 1:**
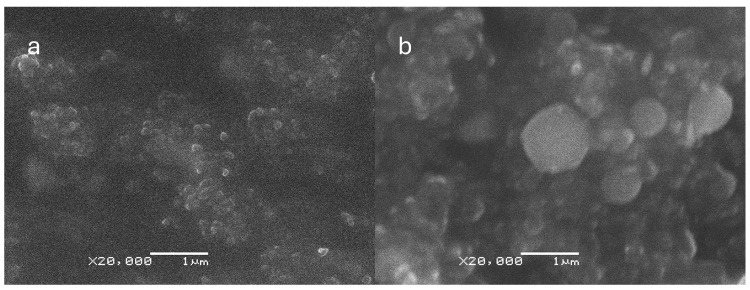
Scanning electron microscopy (SEM) micrographs of (**a**) CNp and (**b**) CNpN. The size bar corresponds to 1000 nm.

The SEM micrographs provided valuable insights into the characteristics of the CNp and CNpN. Specifically, the SEM analysis indicated that CNp exhibited a mean size of approximately 128 ± 20 nm, whereas CNpN displayed a larger size distribution, with an average diameter of around 346 ± 69 nm (Figure 1). These observations align with the trends observed in dynamic light scattering (DLS) analysis, where an increase in nanoparticle size was noted upon the addition of nobiletin. However, it is noteworthy that the sizes determined through SEM were consistently smaller than those measured via DLS. This disparity can be attributed to the dehydration process involved in SEM sample preparation, which often leads to sample shrinkage and thus an underestimation of particle size.

Moreover, the SEM analysis of CNpN provided valuable insights into the morphology of the nanoparticles. Specifically, it revealed a high degree of sphericity, indicating a uniform and well-defined structure. This enhanced understanding of nanoparticle morphology is crucial for elucidating their behavior and interactions in biological and environmental systems.

These findings are consistent with those of prior research [14], thereby reinforcing the reproducibility and reliability of the synthesized nanoparticles. By employing complementary techniques such as SEM and DLS, a comprehensive characterization of the nanoparticles can be achieved, facilitating their potential application in various fields ranging from drug delivery to environmental remediation.

### 3.3. FTIR Spectra, Chitosan–Nobiletin Interaction

**Figure 2:**
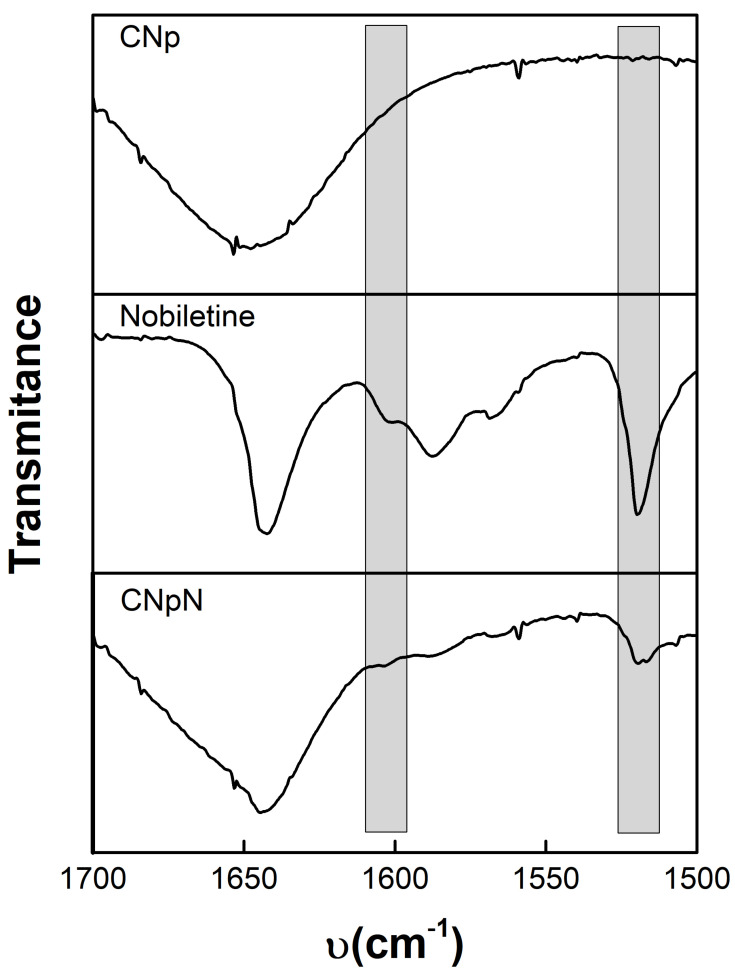
Infrared spectroscopy of: CNp, nobiletin, and CNpN.

Figure 2 depicts the infrared spectroscopy of CNp, nobiletin, and CNpN. The CNp FTIR spectra reveal the existence of dual peaks within the range of 1660–1620 cm−^1^, attributed to the C=O, confirming the presence of residual N-acetyl groups [26,27,28]. The splitting of this peak into distinct bands at 1655 cm−^1^ and approximately 1620 cm−^1^ results from the influence of hydrogen bonding within the polymeric matrix. Additionally, the absorption peak at approximately 1553 cm−^1^ corresponds to the N-H bond of the amide II structure within the polymer. Furthermore, the peak observed at 1652 cm−^1^ is associated with amide I absorption, while the peak at 1597 cm−^1^ originates from the amide II functionality present in the chitosan polymer [29].

Nobiletin exhibits a pair of characteristic signals at 1588 and 1519 cm^−1^, corresponding to the stretching of the aromatic rings of the flavonoid [14]. Notably, this signal was also discernible for CNpN. In the CNpN spectrum, the band present at 1634 cm^−1^ can be associated with the probable imine bond formation between the amino groups of chitosan and the carbonyl group of nobiletin through the Schiff reaction [14]. One of the most simplified reactions of amine groups is the formation of Schiff base (-N=C) via the interaction with carbonyl groups [30].

### 3.4. Effect of CNp and CNpN Treatments on the Viability of S. cerevisiae

**Figure 3:**
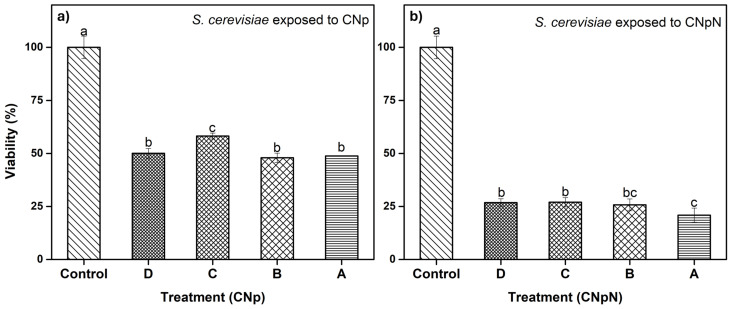
(**a**) Effect of CNp and (**b**) CNpN on the viability of *S. cerevisiae*. Different letters (a–c) indicate a significant difference (*p* < 0.05).

In Figure 3a, we can observe the effect of different treatments on the viability of *S. cerevisiae*. The graph demonstrates that treatments A, B, and D were statistically similar and more effective, as they decreased cell viability, reducing metabolic activity to approximately 50%. In the case of treatment C, metabolic activity was reduced to approximately 60%, making it slightly less effective than the other treatments. In Figure 3b, we can observe the effect on the viability of *S. cerevisiae* under CNpN treatment. It is evident that the viability of *S. cerevisiae* was significantly reduced with all treatments. Treatments C and D were statistically similar, reducing metabolic activity to approximately 25%. Treatment A was the most effective, reducing viability to 20%, while treatment B fell statistically between treatment A and treatments C and D.

### 3.5. Effect of CNp and CNpN Treatments on the Viability of C. albicans

**Figure 4:**
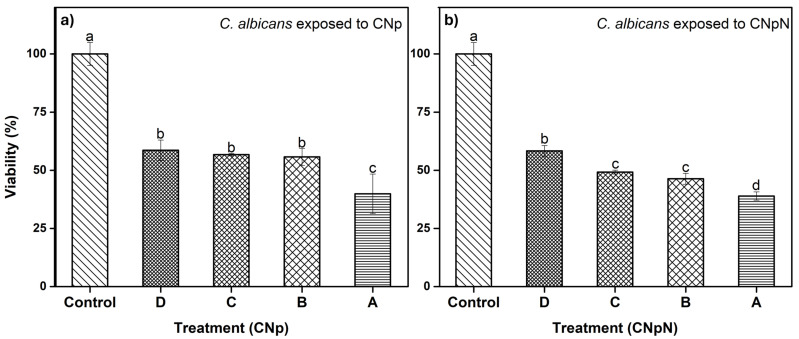
(**a**) Effect of CNp and (**b**) CNpN on the viability of *C. albicans*. Different letters (a–d) indicate a significant difference (*p* < 0.05).

In Figure 4a, we can observe the effect of CNp and CNpN on *C. albicans*. We note that treatments B, C, and D were statistically similar, reducing viability to approximately 60%. Conversely, treatment A was more efficient, reducing the viability of *C. albicans* to approximately 40%. The XTT assay has been employed in investigations involving various fungal species. In a study by Chavez-Magdaleno et al. (2018) [31], it was observed that CS-PEO-Np (80 μg/mL), prepared via nanoprecipitation, resulted in a 90% decrease in the viability of *Colletotrichum gloeosporioides*. Similarly, Luque-Alcaraz et al. (2016) investigated the effects of CS-PEO-Np (200 μg/mL), also synthesized through nanoprecipitation, on the germination of Aspergillus parasiticus spores, reporting a 50% reduction [25]. This decline was attributed to the electrostatic interactions, such as van der Waals forces and hydrogen bonds, occurring between the positive charges of the nanoparticles and the negatively charged groups on the fungal cell membrane. These interactions induced internal instability, altering the membrane’s conformation and ultimately leading to cell death [32]. It is important to note that these treatments consisted solely of CNp, without the presence of nobiletin. Lastly, in Figure 4b, we can observe the effect of CNpN on the viability of *C. albicans*. The least efficient treatment was D, reducing viability to 60%. Treatments B and C were statistically similar, inhibiting 50% of the metabolic activity of the strain, while treatment A reduced viability to 40%, making it the most efficient treatment.

### 3.6. Effect of CNp and CNpN Treatments on the Cell Membrane Integrity of S. cerevisiae

**Figure 5:**
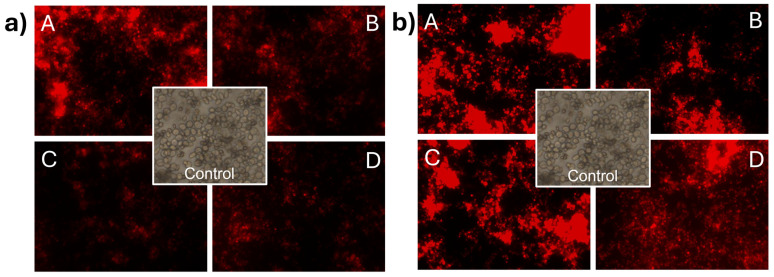
Damage to the cell membrane integrity of *S. cerevisiae* under CNp (**a**) and CNpN (**b**) treatments with different concentrations A, B, C, and D.

In Figure 5a, the impact of CNp treatments on the membrane integrity of *S. cerevisiae* can be observed. The treatments used were (A) 0.08 mg/mL, (B) 0.04 mg/mL, (C) 0.02 mg/mL, and (D) 0.01 mg/mL. Membrane damage was evident as cells were stained with propidium iodide. It can be observed that at concentration A, the intensity of the light emitted by propidium iodide was higher than in the other treatments, indicating that at higher concentrations, more cells lost the integrity of their cell membrane. In the case of *S. cerevisiae* (Figure 5b) treated with CNpN, damage to the membrane integrity was evident with propidium iodide. The luminescent intensity emitted by propidium iodide indicated that the damage to membrane integrity is directly proportional to the concentration of the treatments used.

### 3.7. Effect of CNp and CNpN Treatments on the Cell Membrane Integrity of C. albicans

**Figure 6:**
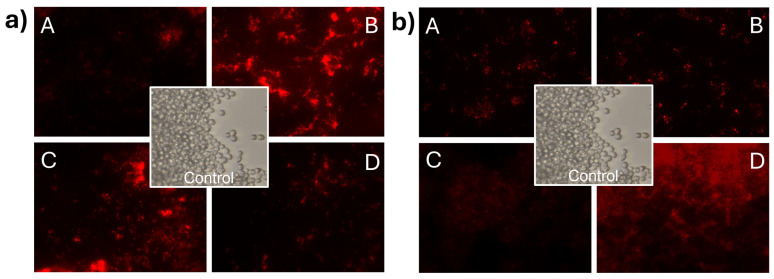
Damage to the cell membrane integrity of *C. albicans* under CNp (**a**) and CNpN (**b**) treatment with different concentrations A, B, C, and D.

In Figure 6a, *C. albicans* cells stained with propidium iodide can be observed, demonstrating membrane integrity damage in *C. albicans* when exposed to CNp treatments. In this case, it can be observed that the intensity of light at the highest concentration was lower than that in *S. cerevisiae*. This phenomenon can be attributed to the inhibition of cell development induced by CNp in treatment A. In contrast, treatments B, C, and D appeared to promote cell viability. However, it is important to note that despite treatments B, C, and D prompting cell viability, membrane integrity damage was still evident when analyzing the viability of the treated cells. The damage effect on the membrane of *C. albicans* (Figure 6b) was even more apparent in the presence of CNpN, where treatments A, B, and C were highly effective in inhibiting cell viability and causing membrane integrity damage. Conversely, in treatment D, there was greater cell viability; however, it was evident that they were stained with propidium iodide.

Observing the images of the fungus stained with propidium iodide revealed visible membrane damage. When the plasma membrane is altered, propidium iodide penetrates the cell interior, reacts, and intercalates with 4–5 base pairs of DNA, emitting high red fluorescence [33]. It has been reported that chitosan exhibits antifungal activity at both low and high concentrations, attributed to various mechanisms of action [34]. At low concentrations, destabilization of ion homeostasis and metabolism has been proposed. An imbalance in the concentrations of K^+^ and Ca^2+^ inside and outside of the cells compromises the integrity of the cell membrane [35]. On the other hand, at high concentrations, mechanisms of action involve damage caused by the destabilization of permeability exerted by electrostatic interactions between positively charged chitosan groups (NH_3_^+^) and negatively charged molecules on the fungal cell surface [36,37]. Nevertheless, multiple internalization mechanisms have been proposed, suggesting that the interaction occurs through various pathways [38].

## 4. Conclusions

Based on the obtained results, it can be concluded that CNpN exhibit significant therapeutic potential in terms of their toxicity and cellular internalization capacity in eukaryotic cell models (*S. cerevisiae* and *C. albicans*). Physicochemical characterization revealed that CNpN exhibit a larger hydrodynamic diameter than CNp, suggesting successful loading of nobiletin in CNpN. Furthermore, SEM imaging showed high sphericity in CNpN, supporting the stability and uniformity of these nanoparticles. Analysis of the ζ-potential demonstrated that both CNp and CNpN possess a positive ζ-potential, indicative of their colloidal stability and ability to prevent aggregation. These findings suggest that CNpN have the potential to be utilized in a wide range of biomedical applications, including in drug delivery and targeted therapy. Additionally, the results of the cellular viability assays and the membrane integrity damage support the notion that CNpN are internalized by the studied eukaryotic cells, indicating their ability to penetrate cells and exert specific biological effects. This encourages further studies to evaluate their efficacy and safety in more complex biological environments, as well as to optimize their properties for specific applications. Collectively, these results support the ongoing interest in the development and application of chitosan nanoparticles loaded with nobiletin as a promising strategy in antifungal and anticancer therapy.

## Figures and Tables

**Table 1:** Serial dilution process to obtain treatments A, B, C, and D.

Treatment	CNp	CNpN	Concentration
A	418.8 μL CNP + 4581.15 mL PDB	1.29 mL CNpN + 3.71 mL PDB	0.16 mg/mL
B	2.5 mL de A + 2.5 mL PDB	2.5 mL de A + 2.5 mL PDB	0.08 mg/mL
C	2.5 mL de B + 2.5 mL PDB	2.5 mL de B + 2.5 mL PDB	0.04 mg/mL
D	2.5 mL de C + 2.5 mL PDB	2.5 mL de C + 2.5 mL PDB	0.02 mg/mL
Control	5 mL PDB	5 mL PDB	0.00 mg/mL

**Table 2:** Hydrodynamic diameter and ζ-potential of CNp and CNpN.

	CNp	CNpN
Hydrodynamic diameter (nm)	288.74 ± 2.37	596.60 ± 35.49
ζ-Potential (mV)	34.51 ± 0.66	37.73 ± 0.19

## Data Availability

The data presented in this study are available on request from the corresponding author.
